# Association between Sarcopenia and Omega-3 Polyunsaturated Fatty Acid in Patients with Hepatocellular Carcinoma

**DOI:** 10.31662/jmaj.2022-0037

**Published:** 2022-03-25

**Authors:** Shinji Itoh, Yoshihiro Nagao, Kazutoyo Morita, Takeshi Kurihara, Takahiro Tomino, Yukiko Kosai-Fujimoto, Noboru Harada, Nobuhiro Fujita, Yasuhiro Ushijima, Masaki Mori, Tomoharu Yoshizumi

**Affiliations:** 1 Department of Surgery and Science, Graduate School of Medical Sciences, Kyushu University, Fukuoka, Japan; 2 Department of Clinical Radiology, Graduate School of Medical Sciences, Kyushu University, Fukuoka, Japan; 3 Tokai University School of Medicine, Isehara, Japan

**Keywords:** sarcopenia, hepatocellular carcinoma, eicosapentaenoic acid, docosahexaenoic acid

## Abstract

**Introduction:**

This study aimed to validate whether preoperative sarcopenia can predict long-term outcomes in patients with hepatocellular carcinoma (HCC) and identify the associations between sarcopenia and polyunsaturated fatty acids (PUFAs).

**Methods:**

This large, retrospective study included 353 patients who underwent hepatic resection for HCC and preoperative computed tomography scans. Skeletal muscle mass was measured at the third lumbar vertebrae. The cutoff value for sarcopenia followed the Japan Society of Hepatology’s assessment criteria for sarcopenia.

**Results:**

Ninety-three patients (26.3%) with preoperative sarcopenia were enrolled. These patients had a significantly lower body mass index (p < 0.0001) and serum albumin level (p = 0.0070) as well as a higher rate of advanced-stage cancer (p = 0.0062) than those without sarcopenia. Patients with sarcopenia had significantly shorter overall survival than the other patients before (p = 0.0001) and after (p = 0.0415) propensity score matching. The sarcopenia group was significantly associated with low levels of eicosapentaenoic acid (EPA) and docosahexaenoic acid (DHA), which were categorized based on omega-3 PUFAs, compared with those in the non-sarcopenia group (p = 0.0030 and p = 0.0135).

**Conclusions:**

We demonstrated an association between sarcopenia and the long-term prognosis in patients with HCC. Low EPA and DHA levels were associated with preoperative sarcopenia. Further prospective studies are needed to investigate whether nutritional support using omega-3 PUFAs can prevent and manage skeletal muscle mass depletion.

## Introduction

Hepatocellular carcinoma (HCC) is a major primary liver malignancy and one of the most common causes of cancer-related deaths worldwide ^(1)^. Hepatic resection is one of the most effective treatments for liver malignancies, including HCC ^(2), (3)^. HCC arises from a background of liver disease caused by hepatitis B virus, hepatitis C virus, alcoholic fatty liver disease, nonalcoholic fatty liver disease, or metabolic-associated fatty liver disease ^(4), (5)^. Therefore, patients with HCC comprise a heterogeneous population in terms of background factors and liver function.

Sarcopenia, defined as a decrease in skeletal muscle mass (SMM) and strength over time, is linked to a higher risk of physical impairment, poor quality of life, and mortality ^(6)^. There is a link between sarcopenia, identified by SMM, and a poor prognosis in patients with cancer ^(7), (8), (9)^. We previously associated preoperative sarcopenia and postoperative SMM loss with a poor outcome after hepatic resection with HCC ^(10), (11), (12)^. The assessment criteria for sarcopenia in liver disease was published by the Japan Society of Hepatology ^(13)^. For nutrition and, especially, for essential fatty acids, there is an association between SMM and omega-3 polyunsaturated fatty acids (PUFAs), including eicosapentaenoic acid (EPA) and docosahexaenoic acid (DHA) ^(14), (15), (16)^. However, to date, SMM in patients with HCC and its association with PUFAs have not been fully examined.

This study aimed to validate the assessment criteria for sarcopenia in liver disease for outcomes in patients with HCC following hepatic resection and to investigate the association between SMM and PUFAs.

## Materials and Methods

### Patients

Three hundred fifty-three patients with HCC who underwent liver resection at the Department of Surgery and Science, Kyushu University hospital, between May 2014 and December 2019, were screened and enrolled, and their medical records were reviewed retrospectively. The study protocol complied with the Code of Ethics of the World Medical Association (Declaration of Helsinki) and the institutional review board (approval numbers: 2021-268).

### Imaging and assessment of SMM

Preoperative computed tomography (CT) scans were used to determine the degree of SMM, as previously reported ^(10), (11)^. A transverse CT image in the inferior direction of the third lumbar vertebra was evaluated on each scan. Thresholds of −29 to +150 HU were used to identify and quantify skeletal muscle (water and air are defined as 0 and 1000 HUs, respectively). The psoas, erector spinae, quadratus lumborum, transversus abdominis, external and internal abdominal oblique muscles, and rectus abdominis were measured. Water and air were used to calibrate CT data at regular intervals. The SMM was measured by manual outlining on CT images. The SMM was standardized using the following formula: (cross-sectional area of the total skeletal muscle at the third lumbar vertebra level in cm^2^)/(height [m] × height [m]). The following previously published formula was used to determine preoperative sarcopenia: <42 cm^2^/m^2^ for men and <38 cm^2^/m^2^ for women ^(13)^.

### Surgical procedures

Our surgical procedures and indications for hepatic resection due to HCC have been reported previously ^(11)^. The plane of transection was marked using intraoperative ultrasonography. The Cavitron Ultrasonic Surgical Aspirator system (Valleylab Inc., Boulder, CO, USA) and a monopolar dissecting sealer (TissueLink; Salient Surgical Technologies, Portsmouth, NH, USA) powered by a VIO system (VIO 300D; ERBE Elektromedizin, Tubingen, Germany) were used to perform a parenchymal transection ^(17)^. The Pringle technique was used to manage inflow vascular control, with 15 minutes of occlusion followed by 5 minutes of reperfusion. Postoperative complications were categorized using the Clavien-Dindo classification ^(18)^. Postoperative complications at 30 days after hepatic resection were classified as grade ≥3a.

### Statistical analysis

Continuous variables were presented as the median and were compared using the Mann-Whitney U test. Categorical variables were reported as percentages and compared using the χ^2^ test or Fisher’s exact test. The cumulative overall survival (OS) rate was calculated using the Kaplan-Meier method, and differences between the curves were evaluated using the log-rank test. Differences were considered significant at p < 0.05. Propensity score matching was performed to overcome the confounding effects of these differences between the sarcopenia and nonsarcopenia groups. Correlations between the SMM and PUFAs in preoperative blood samples were assessed using Spearman’s correlation coefficient test. All statistical analyses were performed using JMP software (SAS Institute Inc., Cary, NC, USA).

## Results

### SMM in patients with HCC, clinicopathological characteristics, and outcome

The median preoperative SMM values in the 353 patients were 47.44 cm^2^/m^2^ (interquartile range [IQR], 42.76-52.29 cm^2^/m^2^) for men and 40.14 cm^2^/m^2^ (IQR, 36.60-44.82 cm^2^/m^2^) for women. The rate of sarcopenia was 22.6% in men and 34.9% in women.

The preoperative clinicopathological characteristics for all patients are shown in Table 1. The following significant differences were found in the sarcopenia group compared with the nonsarcopenia group: older age (p = 0.0011); more female participants (p = 0.0168); a lower body mass index (p < 0.0001); a lower rate of hepatitis B surface antigen positivity (p = 0.0081); low levels of serum albumin (p = 0.0070), total bilirubin (p = 0.0326), indocyanine green clearance test at 15 minutes (p = 0.0300), and total lymphocyte count (p = 0.0229); higher levels of serum alpha-fetoprotein (p = 0.0001) and des-gamma-carboxyprothrombin (p = 0.0006) as well as a larger tumor (p = 0.0042); and a higher rate of Barcelona Clinic Liver Cancer staging B or C (p = 0.0062). There was no significant difference in the serum total cholesterol level between the two groups. For perioperative and pathological characteristics, the sarcopenia group showed a longer postoperative hospital stay (p = 0.0096) and a higher rate of microscopic intrahepatic metastasis (p = 0.0242) in HCC than the nonsarcopenia group (Table 2).

**Table 1.** Background Characteristics of Patients Who Underwent Liver Resection before and after Propensity Score Matching.

Factors	Before propensity score matching			After propensity score matching		
	Nonsarcopenia (n = 260)	Sarcopenia (n = 93)	*P*-value	Nonsarcopenia (n = 72)	Sarcopenia (n = 72)	*P*-value
Age (years)	70 (65-76)	74 (68-78)	0.0011	74 (68-79)	74 (68-77)	0.5485
Sex, male/female	191/69	56/37	0.0168	45/27	45/27	1.0000
BMI (kg/m^2^)	23.9 (22.3-26.2)	20.8 (19.3-23.0)	<0.0001	22.1 (20.3-23.6)	21.8 (19.7-23.5)	0.4745
Diabetes mellitus (%)	100 (38.4%)	36 (38.7%)	0.9663	29 (40.2%)	27 (37.5%)	0.7324
Hypertension (%)	152 (58.4%)	60 (64.5%)	0.3063	43 (59.7%)	47 (65.2%)	0.4911
HBs-Ag positive (%)	54 (20.7%)	8 (8.6%)	0.0081	5 (6.9%)	8 (11.1%)	0.3830
HCV-Ab positive (%)	105 (40.3%)	45 (48.3%)	0.1803	35 (48.6%)	35 (48.6%)	1.0000
Total cholesterol (mg/dl)	175 (151-193)	168 (141-193)	0.2389	175 (151-192)	171 (143-193)	0.4047
Albumin (g/dl)	4.1 (3.8-4.4)	4.0 (3.7-4.2)	0.0070	4.0 (3.6-4.2)	4.1 (3.8-4.2)	0.7362
Total bilirubin (mg/dl)	0.9 (0.7-1.1)	0.8 (0.6-1.0)	0.0326	0.8 (0.6-1.1)	0.8 (0.6-1.0)	0.6368
Prothrombin time (%)	93 (85-102)	93 (86-103)	0.9674	95 (87-105)	94 (86-105)	0.7220
Platelet (×10^4^/μl)	15.6 (11.9-19.2)	16.8 (13.5-22.7)	0.0272	16.6 (12.5-20.9)	16.7 (13.1-20.5)	0.7675
ICGR15 (%)	11.4 (7.8-18.4)	9.5 (5.5-16.1)	0.0300	10.1 (6.6-18.4)	8.5 (4.7-14.9)	0.0817
Child-Pugh classification, grade B	11 (4.2%)	7 (7.5%)	0.2149	2 (2.7%)	5 (6.9%)	0.4414
ALBI, grade 2	102 (39.2%)	43 (46.2%)	0.2386	32 (44.4%)	32 (44.4%)	1.0000
Total lymphocyte count (/mm^3^)	1465 (1131-1898)	1291 (923-1698)	0.0229	1471 (1082-1789)	1379 (950-1780)	0.7162
C-reactive protein (mg/dl)	0.10 (0.04-0.18)	0.09 (0.04-0.22)	0.8250	0.10 (0.04-0.25)	0.09 (0.04-0.20)	0.4764
AFP (ng/ml)	5.2 (3.3-18.7)	14.2 (3.9-388)	0.0001	5.9 (3.1-61.3)	12.8 (3.9-174)	0.2369
DCP (mAU/ml)	37 (22-156)	80 (28.5-1648)	0.0006	96 (26-1548)	77 (29-1145)	0.7745
Tumor size (cm)	2.5 (1.6-4.0)	3.2 (1.8-6.7)	0.0042	3.5 (2.2-6.0)	2.8 (1.7-5.5)	0.2327
Solitary/multiple	202/58	63/30	0.0570	54/18	50/22	0.4568
Macrovascular invasion (%)	13 (5.0%)	6 (6.4%)	0.6001	4 (5.5%)	3 (4.1%)	0.6964
BCLC staging, B or C (%)	35 (13.4%)	24 (25.8%)	0.0062	15 (20.8%)	15 (20.8%)	1.0000
Gross classification, single nodular type (%)	189 (73.5%)	63 (68.4%)	0.3523	48 (66.6%)	51 (70.8%)	0.5896

The data are presented as n (%) or median (interquartile range). BMI, body mass index; HBs-Ag, hepatitis B surface antigen; HCV-Ab, hepatitis C virus antibody; ICGR15, indocyanine green clearance test at 15 minutes; ALBI, albumin-bilirubin; AFP, alpha-fetoprotein; DCP, *des*-gamma-carboxyprothrombin; BCLC, Barcelona Clinic Liver Cancer

**Table 2.** Perioperative and Pathological Characteristics of Patients Who Underwent Liver Resection before and after Propensity Score Matching.

Factors	Before propensity score matching			After propensity score matching		
	Nonsarcopenia (n = 260)	Sarcopenia (n = 93)	*P*-value	Nonsarcopenia (n = 72)	Sarcopenia (n = 72)	*P*-value
Anatomical liver resection (%)	109 (41.9%)	48 (51.6%)	0.1066	32 (44.4%)	32 (44.4%)	1.0000
Major liver resection (%)	29 (11.1%)	16 (17.2%)	0.1332	16 (22.2%)	8 (11.1%)	0.0736
Laparoscopic liver resection (%)	102 (39.2%)	29 (31.1%)	0.1680	21 (29.1%)	20 (27.7%)	0.8535
Duration of surgery (min)	228 (168-284)	247 (166-323)	0.2534	240 (176-277)	238 (149-297)	0.7951
Blood loss (g)	220 (100-500)	250 (126-800)	0.0859	291 (100-536)	231 (82-652)	0.9649
Postoperative complications (%)	35 (13.4%)	16 (17.2%)	0.3783	8 (11.1%)	14 (19.4%)	0.1646
Postoperative hospital stay (days)	10 (8-13)	11 (9-15)	0.0096	11 (8-14)	11 (8-15)	0.4162
Poor differentiation (%)	66 (25.8%)	34 (36.5%)	0.0515	23 (32.3%)	26 (36.1%)	0.6396
Microscopic vascular invasion (%)	44 (17.1%)	20 (21.5%)	0.3486	17 (23.6%)	12 (16.6%)	0.2988
Microscopic intrahepatic metastasis (%)	39 (15.2%)	24 (25.8%)	0.0242	11 (15.2%)	17 (23.6%)	0.2065
F3 or F4 (%)	99 (38.0%)	33 (36.2%)	0.7586	19 (26.3%)	27 (38.5%)	0.1209

The data are presented as n (%) or median (interquartile range). F, fibrosis of the liver

Propensity score matching was used to overcome the confounding effects of these differences between the two groups. After propensity score matching, there were no significant differences in the pre- and perioperative and pathological characteristics between the two groups (Table 1 and 2).

**Figure 1.**
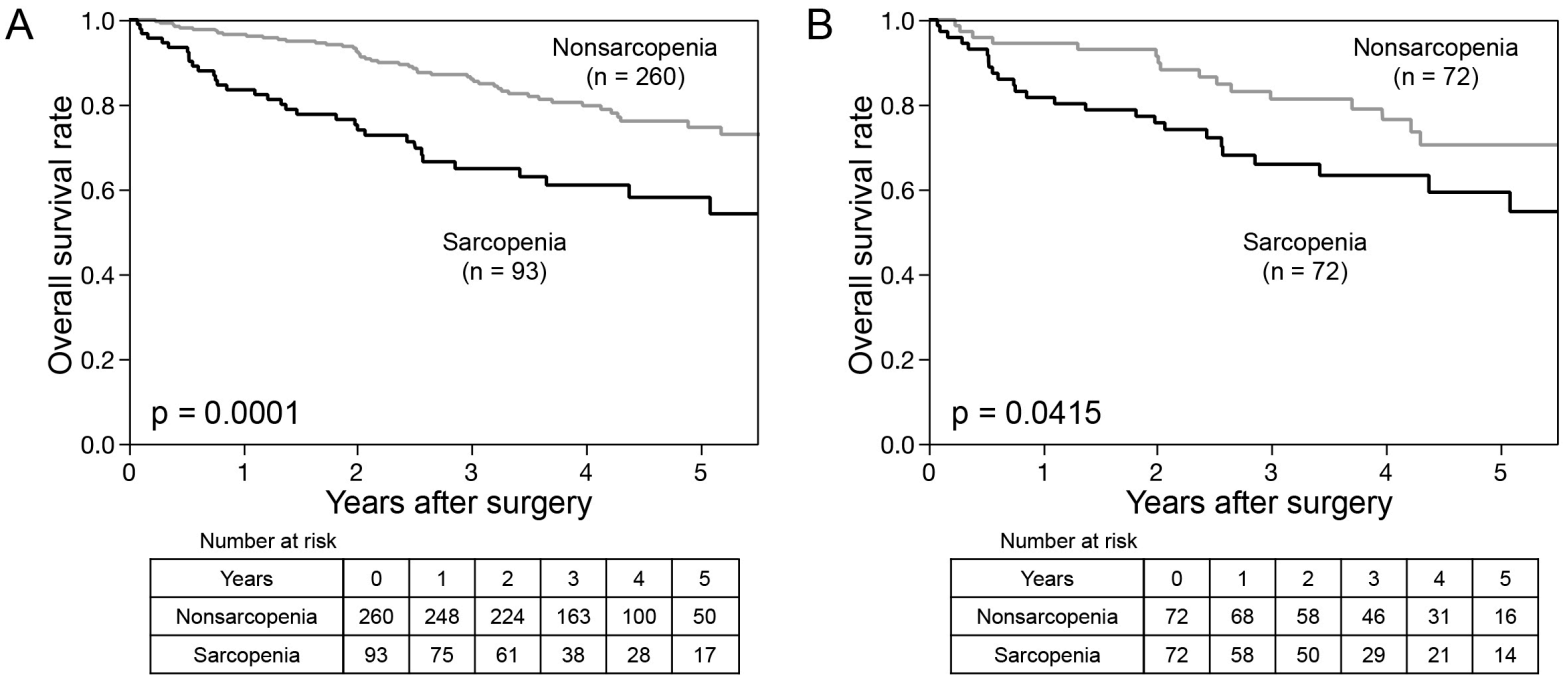
Kaplan-Meier curves for overall survival in patients with hepatocellular carcinoma on the basis of sarcopenia.

The Kaplan-Meier curves are shown in Figure 1. Patients with sarcopenia had a significantly worse prognosis for OS than those with nonsarcopenia before and after propensity score matching analysis (p = 0.0001 and p = 0.0415, respectively).

### Association of SMM with PUFAs

**Figure 2.**
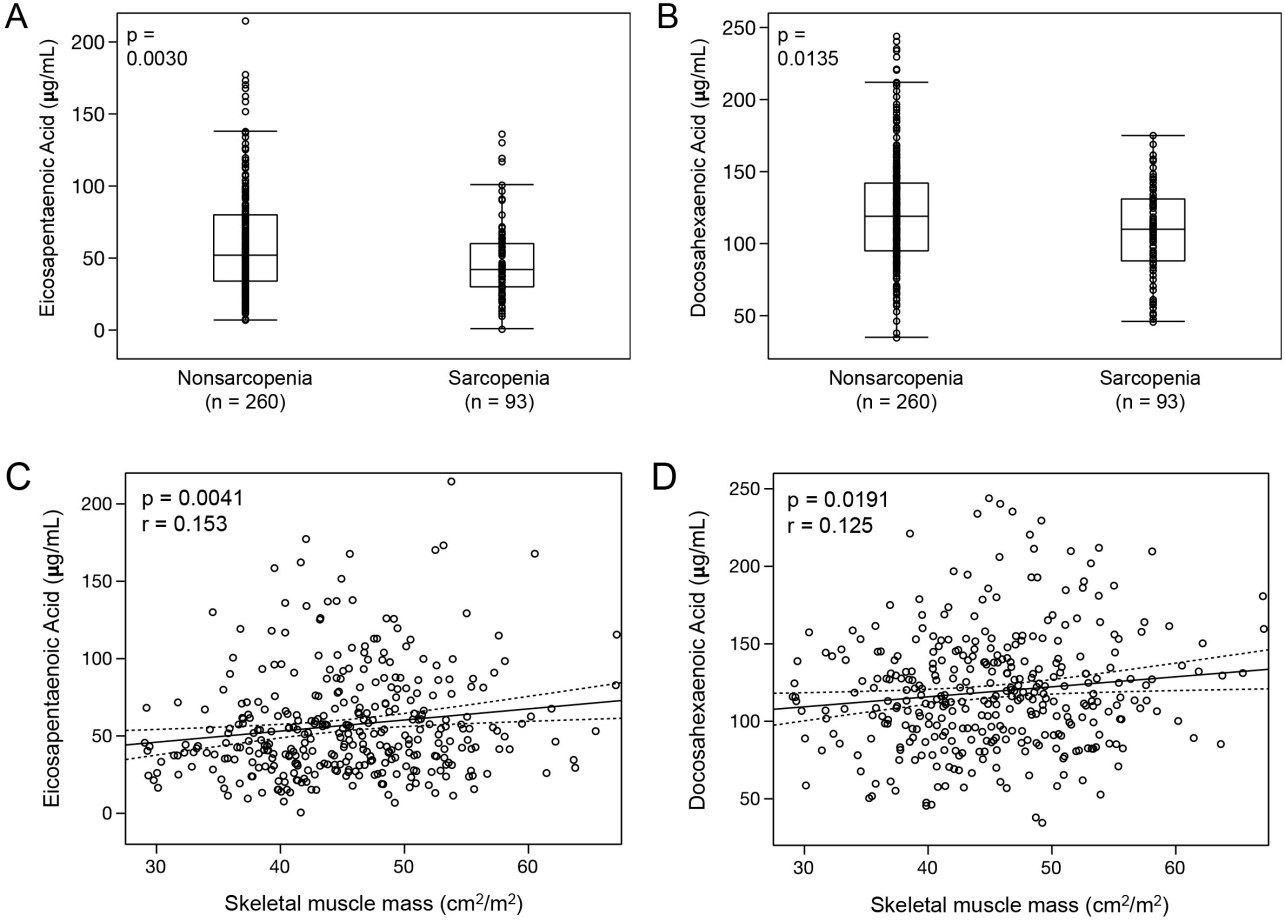
Distributions of eicosapentaenoic acid and docosahexaenoic acid on the basis of skeletal muscle mass as a categorical (A and B) or continuous (C and D) variable.

For PUFAs, the median EPA, DHA, arachidonic acid (AA), and dihomo-gamma-linolenic acid (DGLA) levels in all patients were 48.6 μg/mL (IQR, 32.6-71.6 μg/mL), 115.5 μg/mL (IQR, 92.2-139.4 μg/mL), 155.3 μg/mL (IQR, 132.2-184.4 μg/mL), and 31.2 μg/mL (IQR, 24.9-37.9 μg/mL), respectively. The sarcopenia group was significantly associated with low EPA and DHA levels compared with those in the nonsarcopenia group (EPA in sarcopenia: median, 42.0 μg/mL [IQR, 29.8-60.4 μg/mL] vs. EPA in nonsarcopenia: 52.3 μg/mL [IQR, 33.8-80.3 μg/mL], p = 0.0030, Figure 2A; DHA in sarcopenia: median, 109.8 μg/mL [IQR, 87.7-131.4 μg/mL] vs. DHA in nonsarcopenia: median, 118.7 μg/mL [IQR, 94.7-142.5 μg/mL], p = 0.0135, Figure 2B). Spearman’s correlation coefficient analysis also showed a positive correlation between the SMM and EPA or DHA (EPA: Spearman’s rho = 0.153, p = 0.0041, Figure 2C; DHA: Spearman’s rho = 0.125, p = 0.0191, Figure 2D). However, there was no significant difference in AA between the two groups (AA in sarcopenia: median, 154.2 μg/mL [IQR, 128.2-184.0 μg/mL] vs. AA in nonsarcopenia; median, 155.4 μg/mL [IQR, 133.9-184.4 μg/mL], p = 0.2481). The DGLA level in the sarcopenia group tended to be low compared with that of the nonsarcopenia group (DGLA in sarcopenia: median, 29.9 μg/mL [IQR, 22.2-36.0 μg/mL] vs. DGLA in nonsarcopenia: median, 31.7 μg/mL [IQR, 25.8-38.4 μg/mL], p = 0.0542).

**Figure 3.**
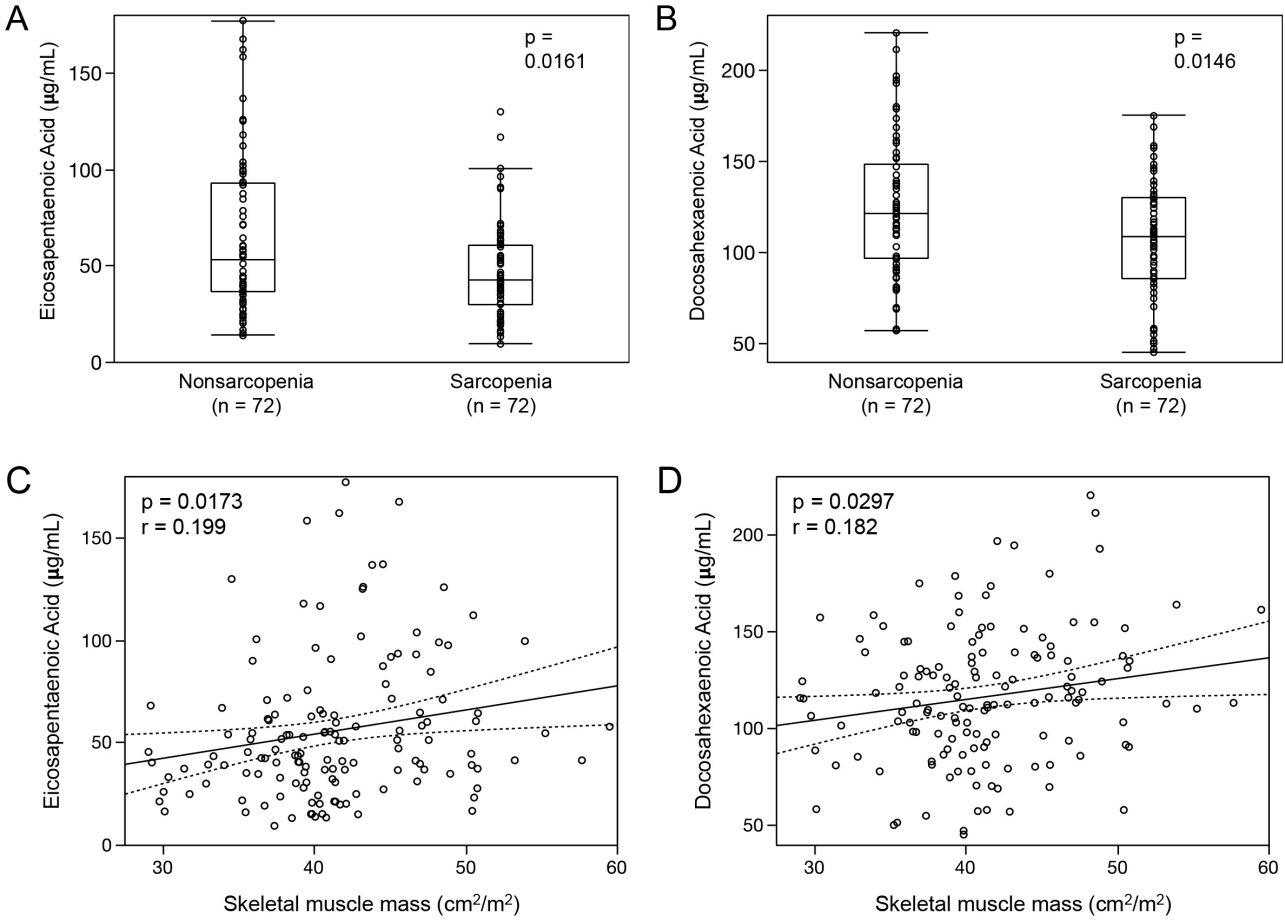
Distributions of eicosapentaenoic acid and docosahexaenoic acid on the basis of skeletal muscle mass as a categorical (A and B) or continuous (C and D) variable after propensity score matching analysis.

After propensity score matching was used to overcome the confounding effects of preoperative baseline differences between the two groups, the sarcopenia group had significantly lower EPA and DHA levels than those in the nonsarcopenia group (EPA in sarcopenia: median, 42.5 μg/mL [IQR, 30.2-42.4 μg/mL] vs. EPA in nonsarcopenia: median, 53.0 μg/mL [IQR, 36.4-93.3 μg/mL], p = 0.0161, Figure 3A; DHA in sarcopenia: median, 109.2 μg/mL [IQR, 85.8-130.5 μg/mL] vs. DHA in nonsarcopenia: median, 121.5 μg/mL [IQR, 96.9-148.2 μg/mL], p = 0.0146, Figure 3B). Spearman’s correlation coefficient analysis also showed a positive correlation between the SMM and EPA or DHA (EPA: Spearman’s rho = 0.199, p = 0.0173, Figure 3C; DHA: Spearman’s rho = 0.182, p = 0.0297, Figure 3D). However, the difference in AA and DGLA between the two groups was not significant (AA in sarcopenia: median, 156.5 μg/mL [IQR, 128.8-185.5 μg/mL] vs. AA in nonsarcopenia: median, 163.9 μg/mL [IQR, 138.4-183.9 μg/mL], p = 0.2939; DGLA in sarcopenia: median, 30.9 μg/mL [IQR, 25.2-36.5 μg/mL] vs. DGLA in nonsarcopenia: median, 29.7 μg/mL [IQR, 24.1-37.0 μg/mL], p = 0.5432).

## Discussion

This study revealed that preoperative sarcopenia, defined using the Japan Society of Hepatology guidelines for sarcopenia in liver disease ^(13)^, was significantly associated with poor survival in patients with HCC following hepatic resection. Moreover, sarcopenia in patients with HCC was correlated with low EPA and DHA levels before and after propensity score matching analysis.

Several studies have reported that sarcopenia affected postoperative prognosis for patients with HCC following hepatic resection. Voron et al. reported that sarcopenia was a strong and independent prognostic factor for mortality after hepatectomy for 198 European patients with HCC, which was defined as follows: SMM <52.4 cm^2^/m^2^ for men and <38.9 cm^2^/m^2^ for women ^(19)^. Kobayashi et al. showed that sarcopenic obesity was associated with worse outcomes following hepatectomy in 465 Japanese patients with HCC, and sarcopenia cutoff values for SMM were 40.31 cm^2^/m^2^ for men and 30.88 cm^2^/m^2^ for women ^(20)^. In our previous reports ^(10), (11)^, sarcopenia was defined as follows: <43.75 cm^2^/m^2^ for men and <41.10 cm^2^/m^2^ for women. There were no significant differences in tumor-related factors or surgical outcomes, except that liver function and sarcopenia were predictive of worse OS. Thus, different cutoff values were used in each study. Therefore, this validation study was conducted using the cutoff values for sarcopenia in liver disease published by the Japan Society of Hepatology ^(13)^. This study showed that preoperative sarcopenia in patients with HCC was associated with tumor-related factors, including tumor markers, size, Barcelona Clinic Liver Cancer staging, and poor OS before and after propensity score matching analysis. Recent changes ^(21)^, such as the aging of HCC incidence and the increase in HCC that arises from nonalcoholic fatty liver disease or metabolic-associated fatty liver disease, might have influenced the correlation between tumor-related factors and sarcopenia.

The omega-3 PUFAs, EPA and DHA, play important roles in human health, with one of its key functions being to decrease inflammation and facilitate its resolution ^(22)^. Supplementation with omega-3 PUFAs was reported to improve SMM and to be useful in preventing sarcopenia ^(23)^. In a randomized clinical trial, Smith et al. showed that fish oil-derived omega-3 PUFA therapy increased muscle volume and strength compared with the control group in healthy older adults ^(24)^. For patients with cancer, omega-3 fatty acid concentrations were correlated with sarcopenia and muscle loss at baseline and during chemotherapy for lung cancer ^(15)^. Shirai et al. reported that fish oil-enriched nutrition improved SMM and lean body mass in patients with gastrointestinal cancer who received systemic chemotherapy ^(25)^. This study associated preoperative sarcopenia in patients with HCC with low levels of EPA and DHA but not DGLA and AA before and after propensity score matching analysis. To the best of our knowledge, this is the first clinical study to identify the relationship between sarcopenia and omega-3 PUFAs, including EPA and DHA, for patients with HCC.

Nutrition intervention is important for older adults and patients with cancer to prevent and manage SMM depletion ^(26), (27)^. The internationally accepted European Society for Clinical Nutrition and Metabolism practice guidelines on clinical nutrition in cancer recommends nutritional intervention to increase oral intake in patients with cancer who can eat but are malnourished or at risk of malnutrition and to maintain or increase the level of physical activity to support muscle mass, physical function, and metabolic pattern ^(28)^. For liver disease, branched-chain amino acids (BCAAs) are a class of three essential amino acids comprising valine, leucine, and isoleucine that are protein synthesis and ammonia detoxification substrates and a source of energy for skeletal muscle ^(29)^. A recent prospective randomized, double-blind study revealed that combining BCAA supplementation with nutritional and physical activity interventions improved SMM in cirrhotic patients with sarcopenia ^(30)^. Our results suggested that omega-3 PUFAs are candidates for a new nutritional intervention for HCC patients with sarcopenia. Further studies and a clinical trial involving omega-3 PUFAs for patients with HCC are required.

This is the largest retrospective cohort study to focus on preoperative SMM and omega-3 PUFAs in patients with HCC following hepatic resection. We believe that these results are meaningful and reliable for hepatologists treating HCC. However, this study has a limitation. This study was conducted at a single institution and had a retrospective design. The accumulation of clinical data from prospective studies involving multiple institutions could be useful.

In conclusion, this large retrospective study demonstrated that preoperative sarcopenia, assessed using the Japan Society of Hepatology guidelines, was significantly correlated with unfavorable survival in patients with HCC following hepatic resection. Low EPA and DHA levels were associated with preoperative sarcopenia. Further prospective studies are needed to investigate whether nutritional support using omega-3 PUFAs can prevent and manage SMM loss.

## Article Information

This article is based on the study, which received the Medical Research Encouragement Prize of The Japan Medical Association in 2019.

### Conflicts of Interest

None

### Sources of Funding

This work was supported by the Medical Research Encouragement Prize from The Japan Medical Association and by a JSPS KAKENHI grant Number [JP-19K09198]. The funding sources had no role in the collection, analysis, or interpretation of the data or in the decision to submit the article for publication.

### Acknowledgement

We thank Edanz (https://jp.edanz.com/ac) for editing a draft of this manuscript.

### Author Contributions

S.I. participated in study conception and design, data acquisition, analysis, interpretation, and article drafting.

Y.N., K.M., T.K., T.T., Y.K., N.H., N.F., and Y.U. participated in data acquisition.

M.M. and T.Y. participated in the critical revision of the manuscript.

### Disclaimer

Masaki Mori is one of the Associate Editors of JMA Journal and on the journal’s Editorial Staff. He was not involved in the editorial evaluation or decision to accept this article for publication at all.
